# Supplementary material for: Wireless, battery-free, and real-time monitoring of water permeation across thin-film encapsulation
Source: Nat Commun. 2024 Aug 28;15:7443. doi: 10.1038/s41467-024-51247-3 (PMC11358307; doi:10.1038/s41467-024-51247-3)
Supplement: Supplementary file 1 — Supplementary Information File [file 41467_2024_51247_MOESM1_ESM.docx]

Supplementary Material

Wireless, battery-free, and real-time monitoring of water permeation across thin-film encapsulation

Massimo Mariello^1,2,5^, James Daniel Rosenthal^1^, Francesco Cecchetti^3^, Mingxiang Gao^4^, Anja K. Skrivervik^4^, Yves Leterrier^2^, Stéphanie P. Lacour^1^.

^1^ Laboratory for Soft Bioelectronic Interfaces (LSBI), Neuro-X Institute, École Polytechnique Fédérale de Lausanne, Geneva, Switzerland.

^2^ Laboratory for Processing of Advanced Composites (LPAC), École Polytechnique Fédérale de Lausanne (EPFL), Lausanne, Switzerland

^3^ École Polytechnique Fédérale de Lausanne (EPFL), Lausanne, Switzerland.

^4^ Microwaves and Antennas Group (MAG), École Polytechnique Fédérale de Lausanne (EPFL), Lausanne, Switzerland.

^5^ Current address: Institute of Biomedical Engineering, Department of Engineering Science, University of Oxford, OX3 7DQ, Oxford, UK

1. Design and fabrication

**Fabrication of flexible Mg test sensors.** The fabrication process of the flexible test sensors is illustrated in Figure S3(a-p) and starts with the spin-coating of a 5 µm-thick layer of polyimide (HD Microsystems GmbH, catalog no. PI2611) on a 4-inch silicon wafer at 2500 rpm, followed by soft bake (70°C for 3min, 110°C for 3min) and curing for 2 h at 300°C in a N2-filled oven. The interconnection tracks in Ti (20 nm)/Pt (150 nm) were sputtered (Alliance Concept AC450) and patterned through liftoff with a first photolithography (MicroChemicals, catalog no. AZ1512). Mg thin films (200 nm) were thermally evaporated (Alliance Concept E300) and patterned in shape of serpentines or stripes with different widths through a second photolithography-liftoff step. A second 5 µm-thick PI layer was spin coated on top of the Mg patterns and cured in the same way as the first one, then it was patterned through photolithography (MicroChemicals, catalog no. AZ10XT) and RIE-etching (Corial 210IL) to expose the contact pads. Wiring was carried out with sliver paste or low-temperature soldering onto the pads and finally the connections were mechanically fixed with silicone° ped by laser cutting (Optec WS Turret 200) and peeled off from the Si carrier. The same process flow was adopted for the fabrication of the disposable Mg inserts used for the assembly of the flexible implantable system.

Five designs were selected for the patterning of Mg thin films and they are reported in Figure S3(q), whereas Table S1 summarizes the geometrical parameters of each design.

**Table S1.** Selected designs of Mg test sensors for the calibration of the WPS. The length *l* is the longitudinal dimension of the Mg resistor along which the current flows. The Mg thickness was kept constant at ~200 nm.

| **Design** |  | **Length (*l*, mm)** | **Width (*w*, mm)** | **Thickness (*h*, nm)** |
| --- | --- | --- | --- | --- |
| 1 (long narrow serpentine) |  | 90 | 0.1 | 200 |
| 2 (short narrow serpentine) |  | 45 | 0.1 | 200 |
| 3 (wide stripe) |  | 5 | 1.0 | 200 |
| 4 (narrow stripe) |  | 2.5 | 0.1 | 200 |
| 5 (wide serpentine) |  | 37 | 0.5 | 200 |

**Design of resistance-to-frequency conversion system.** The resistance-to-frequency converting wireless backscatter system is based around a variable frequency CMOS square-wave oscillator whose fundamental frequency is tuned by an external resistance. The square-wave oscillator output is used to actuate a single-pole-single-throw (SPST) RF switch such that one of two discrete impedances will be presented to an antenna connected to the RF common (RFC) port of the switch at the oscillating frequency.

The WPS system was designed in Altium (Altium LLC) schematic capture and circuit board layout software using commercial off-the-shelf parts, except for the implantable antennas (see Section S1.3).

We calibrated the WPS system considering two tunable oscillators, i.e. LTC1799 and LTC6902, (Analog Devices). The expression correlating the oscillation frequency $f_{OSC}$ with the set resistor$R_{set}$ can be written as follows:

|  | $f_{OSC}=\frac{\psi\alpha}{R_{\mathrm{set}}}$ | ( 1 ) |
| --- | --- | --- |

where $\alpha=1,2$ respectively for LTC1799 and LTC6902.

$R_{\mathrm{fix}}$ was chosen in the range provided by the oscillators’ manufacturers (20kΩ-400kΩ for LCT6902 and 3kΩ-3MΩ for LTC1799). Using the oscillator LTC1799 allows to achieve a larger $S_{OSC}$ sensitivity as shown in Figure S8, where the sensitivity curves for the two oscillators are reported for an oscillation frequency of 1MHz. Concerning the power consumption, we compared the two oscillators in terms of dissipated power (see Figure S8(c)): the power cost is similar, especially in the 4-6 MHz region, because LTC1799 requires half the value of $R_{fix}$ used for LTC6902, allowing a higher current flow (under the same supply voltage). Collectively, the previous considerations led us select the LTC1799 oscillator for its smaller size and complexity along a higher sensitivity. We opted to perform the measurements with an oscillation frequency within the 3.5-4 MHz range, to achieve sufficiently high sensitivity $S_{OSC}$: as shown in Figure S8(b), correspondingly to such a frequency, a shift in $f_{OSC}$ of ~35 Hz for a resistance change of 1 Ω can be achieved.

The rigid and flexible printed circuit boards (PCBs) were supplied from PCBWay using their standard two-layer PCB processes. PCB assembly was performed using low temperature lead-free solder paste and manually placing components by hand. Soldering was then performed using a hot plate.

**Design of implantable antennas.** To achieve backscatter communication from in-body implants, we designed flexible implantable antennas operating at the ISM band of 2.4 GHz-2.5 GHz.

Due to the high loss characteristics of the biological tissues surrounding the implants, the major limitation of the implanted antenna is their low radiation efficiency, resulting in tight link budgets [1–3]. However, due to the high water content of biological tissues, their high permittivity allows the antenna size to be effectively reduced, as the wavelength is shorter.

Considering the limited volume of the implant, especially the very thin thickness for subcutaneous implants, we adopted the design of a dipole antenna with meander lines (see Figure 5(e) and Figure S14(b)). In order to minimize the near-field losses of the implanted antenna [4] we set the total thickness of the encapsulation around the antenna to 1 mm. For the balanced feed port of the dipole antenna, we introduced a balun between the RF port and the antenna itself. Specifically, a multilayer chip balun (Johanson Technology, 2450BL15K100E) was used to convert the 50-Ω unbalanced RF port to the 100-Ω balanced coplanar stripline.

Another important point is the impedance matching circuit between the antenna and the feed line (i.e., the 100-Ω coplanar stripline). Unlike antennas operating in free space, the input impedance of implanted antennas is significantly affected by the surrounding biological tissues, especially when the dimension of the antenna encapsulation is electrically small. As shown in Figure S14(a), we measured the input impedance of the designed antenna implanted in a cuboid chicken phantom. According to the original impedance of the antenna (see Figure S14(c)), a π-matching network was designed at the center frequency of 2.45 GHz, with the inductor of 4 nH and both capacitors of 1.2 pF (see Figure 5(e)). The input impedance of the antenna after matching was measured using a vector network analyzer (Hewlett Packard, 8720C) and it is shown in Figure S14(c) on a Smith chart. The reflection coefficient of the antenna indicates that it operates from 2.36 GHz to 2.61 GHz, which fully covers the ISM band used. We carried out similar impedance measurements in other body phantoms (such as cadavers of mouse and rat) and found that this matching network performed well in most subcutaneous implantation locations.

**Design of wireless powering unit.** To enable wireless powering of the implantable platform, we designed a wireless powering unit operating at the High Frequency (HF) RFID band of 13.56 MHz. The unit utilizes the inductive (or magnetic) coupling between two coils as the basic principle for wireless power transfer.

The unit consists of an external transmitter coil (or primary coil) connected to a high frequency power source and a receiver coil (or secondary coil) on the implant [5,6]. The transmitter coil is made of copper wire with three turns and has a diameter of 10 cm. Two designs of the receiver coil were used in i-WPS and miniaturized i-WPS, respectively. Both coils have a size of 22 mm × 14 mm, but differ in the number of turns, metal coverlay, line width, and gap distance (see Figure 5(f) and Figure 6(a) for details). The structural design and optimization of the coils were carried out by full-wave simulations using CST Studio Suite. As illustrated in Figure S17(a, b), the surface current distribution on both coils is uniform in both straight lines and bends, at 13.56 MHz. In order to tune the coil to resonate at 13.56 MHz, we simulated the input impedance of the coil in vacuum (see Figure S17(c, d)), which provided a reference value for the shunt capacitor at the terminal of the coil. In practice, the resonant frequency of the coil is related to both the implantation location and the encapsulation thickness, due to the variation of the inter-turn parasitic capacitance. As a result, in the realization of miniaturized i-WPS, a 56-pF capacitor is adopted.

**Fabrication and assembly of flexible i-WPS.** The fabrication process of the flexible test sensors is illustrated in Figure S11. The flexible platform was fabricated as follows. On a 4-inch Si wafer, 10 µm-thick polyimide (HD Microsystems GmbH, catalog no. PI2611) was spin-coated, soft-baked and cured. A metallization of Ti (20 nm)/ Au (5 µm) was performed by reactive sputtering (Alliance Concept AC450) and the patterning was carried out through liftoff, using a thick resist (MicroChemicals, catalog no. AZ10XT). A second layer of polyimide (5 µm) was spin-coated and cured on top of the metallization, and patterned through another photolithography (MicroChemicals, catalog no. AZ10XT) and RIE-etching (Corial 210IL) to expose all the connection points. After, laser-cutting (Optec WS Turret 200), the devices were peeled off. The disposable Mg inserts were fabricated in the same way as the Mg test sensors, but adjusting the design for the space available in the flexible wireless platform. Before assembling the system, the selected encapsulations were deposited on the Mg inserts, protecting the connections pads.

For the assembly, the flexible platform was positioned and fixed with a tape on a glass slide with a PI sheet in between to avoid the attachment of the system to the glass during soldering. Sn/Bi/Ag solder paste (SMDLTLFP10TS, Chipquik) bumps were dispensed manually on the connection pads exposed and afterwards all the electrical components were positioned using a pick-and-place equipment (JFP Microtechnic). A reflow at 200°C of the solder paste ensured the soldering of all the components. The solder paste was also dispensed on the pads of the Mg inserts which were then flipped and aligned with the pads present on the flexible platform. Thus, air-gun soldering was performed. To guarantee a mechanical fixation and protection for all the components of the system, the flexible platform was covered by a layer of silicone and second layer of poly(isobutylene) (PIB, Oppanol, BASF) dissolved in cyclohexane (Sigma-Aldrich) at 20%wt.

**Scanning Electron Microscopy (SEM), Focused-Ion-Beam (FIB) milling and EDX analysis.** SEM images of all the samples were acquired with a Zeiss GeminiSEM 300 microscope using an annular detector at a beam energy of 3 keV. A 4-nm thick gold layer was preliminarily deposited with a Quorum Q300T Sputter Coater to avoid charging effects during SEM observation.

The cross sections of the flexible Mg test vehicles were obtained by FIB milling in a dual beam equipment (Zeiss CrossBeam 540) equipped with Oxford Inst. EDX detector. The cross section was milled with a current of 7 nA using a trapezoidal window and a second polishing step was performed at 1.5 nA using a narrow rectangular window: the milling depth was always in the range 8-10 µm and the milling width was 20 µm. EDX analysis was performed (with 2 pA current and energy range of 20 keV) on the FIB-milled cross sections to obtain a compositional mapping of the chemical elements present.

**Atomic Force Microscopy (AFM).** AFM images of the Mg morphology before, during and after corrosion were acquired with a Bruker FastScan AFM microscope in contact and tapping mode.

**Deposition of standard and hybrid encapsulations.** The standard barrier encapsulations selected for this work (i.e. PI, PDMS and parylene C) were deposited through usual cleanroom processes. PI was part of the fabrication of the Mg test sensors: it was spin-coated, soft-baked at 70°C for 3min and at 110°C for 3 min, then hard-baked in nitrogen environment. PDMS was first prepared by mixing a pre-polymer precursor and a curing agent (ratio 10:1), then it was spin-coated and cured at 95°C for 1h. Before the spin-coating the pads of the Mg test sensors were covered with a tape and this was peeled off before curing. Parylene C was deposited through room-temperature chemical vapour deposition (RT-CVD) following the Gorham route in a Comelec C-30-S machine.

The hybrid multilayer encapsulations were deposited in a single chamber, from Comelec C30H equipment platform. The deposition of the organic layers (Parylene C) occurred at 30°C whereas that for the ALD inorganic layers (Al_2_O_3_ and TiO_2_) was set with a limit temperature of 100°C in order to minimize thermal annealing effects onto the organic films. Nitrogen was used as purge gas together with all the gas precursors. The growth per cycle for Al_2_O_3_ and TiO_2_ were 1.7 and 0.76 Å, respectively. To provide a conformal deposition of the encapsulations on the devices fabricated in this work, they were hung vertically in the chamber.

**Mechanical buckling tests.** The Mg devices were clamped on a home-made uniaxial stretcher and stretched at 10% of their initial length at 1 Hz (1 stretching cycle per second). The electrical resistance was measured using a 4-point probe configuration and a sourcemeter (Keithley 2400). The stretcher and sourcemeter were both controlled using custom made GUI interface based on LabView 2015.

**In vitro experimental characterization.** In order to validate the designed system and evaluate the long-term functionality of the fabricated devices (Mg test sensors), accelerated aging experiments were carried out. Soaking tests in PBS with a pH of 7.4 (1×, Gibco), at different temperatures (37°C, 67°C, 85°C), were performed, using sealed glass containers inserted in a ventilated climatic chamber (ClimeEvent Weisstechnik; Heraeus HC7015) or on a hot plate.

**Real-time resistance and frequency monitoring.** The variation of the Mg electrical resistance was monitored over time. The resistance was monitored by measuring the voltage while applying a current of 1 mA. The interface was controlled and synchronized using a microcontroller and customized software based on LabVIEW 2015 (National Instruments), and it was able to provide a sampling rate of 0.03 Hz for the same Mg structure. In order to protect the contact points between the Mg structures and the connection tracks, a 15 wt% solution of poly(isobutylene) (PIB, Oppanol, BASF) dissolved in cyclohexane (Sigma-Aldrich) was drop casted onto those points via pneumatic printing: after curing at room temperature a thin layer of high-barrier sealant formed which delayed water permeation. Finally, the connections were embedded in epoxy to prevent any failure or damage during soaking. The same procedures were followed for the frequency measurements which were performed simultaneously with the resistance monitoring.

**Ex vivo and in vivo-like experimental characterization.** *Ex vivo* measurements were performed on explanted chicken tissues which were previously submerged into a physiological solution and kept into a refrigerated environment. The measurements consist of positioning the tissue in a PBS bath kept at body temperature (approximately 37°C). The PBS was aimed at keeping the tissue always hydrated. In order to avoid unproper modifications of the mechanical and viscoelastic properties of the tissue and keep them close to the *in vivo*-like properties, before the measurement, it was removed from the refrigerated environment and kept at room temperature until thermal equilibrium. A hot plate was then used to control and monitor the temperature of the fluid from ~25°C to 37°C at a rate of 1°C /3 min. The characterization of the flexible platform followed the same equipment and methods as in vitro.

The *in vivo-like* measurements were performed on mice bodies (adult male or female C57BL/6 mice (body weight 18–35 g, age 8-25 weeks)): for this purpose, before the experiments, animals were deeply anesthetized and killed with intravenous (IV) injection of ketamine-xylazine mixture (150 mg/kg and 10 mg/kg, respectively). The bodies were kept in a chamber with controlled temperature (body temperature, 37°C) during the experiments. The characterization of the implanted flexible platform followed the same equipment and methods as in vitro. In order to make the high frequency power distribution uniform, we also used a coupling coil tuned for the circuit’s resonant frequency with a shunt capacitor. All the procedures adopted (i.e. housing, surgery, recordings and euthanasia) were performed in compliance with the Swiss Veterinary Law guidelines and approved by the Veterinary Office of the Canton of Geneva (see procedures in previous works [7]).

**Reproducibility.** All data are collected as mean ± s.d. unless stated otherwise, and measurements were taken from distinct samples.

2. Theory

**Correlation between WTR and wireless signal: analytical model.** The water (vapour) transmission rate is defined as the time variation of water mass ($m_{H_{2}O}$) permeated through a barrier per unit area:

|  | $WTR=\frac{1}{S}\cdot\frac{dm_{H_{2}O}}{dt}$ | ( 2 ) |
| --- | --- | --- |

where $S$ is the area of the barrier surface permeated by water molecules.

The Mg corrosion reaction is provided here:

|  | $Mg+2H_{2}O\to Mg\left( OH \right)_{2}+H_{2}$ | ( 3 ) |
| --- | --- | --- |

Hence, the amount of permeated water though the barrier ($m_{H_{2}O}$) can be correlated with the amount of Mg mass consumed due to corrosion ($m_{Mg}$), as follows:

|  | $n_{Mg}=\frac{n_{H_{2}O}}{2}\Rightarrow\frac{m_{Mg}}{M_{Mg}}=\frac{m_{H_{2}O}}{2M_{H_{2}O}}\Rightarrow\frac{dm_{H_{2}O}}{dt}=-2\left( \frac{M_{H_{2}O}}{M_{Mg}} \right)\frac{dm_{Mg}}{dt}$ | ( 4 ) |
| --- | --- | --- |

where $n_{Mg},n_{H_{2}O}$ are the number of moles of Mg and water; $M_{Mg},M_{H_{2}O}$ are the molar masses of Mg and water, respectively

Assuming a model of uniform variation of the Mg thickness during corrosion, it is possible to correlate the consumed Mg mass with its thickness and its electrical resistance, hence it can be shown that the WTR can be expressed as follows:

|  | $WTR=K\cdot\frac{1}{R_{Mg}^{2}}\frac{dR_{Mg}}{dt}$ | ( 5 ) |
| --- | --- | --- |

where $K$ is a constant depending only on geometrical and physical parameters of the Mg structure under corrosion (i.e. Mg mass density, Mg electrical resistivity, molar masses of Mg and water, the Mg area exposed to water permeation).

The time variation of the Mg resistance can be expressed as follows:

|  | $\frac{dR_{Mg}}{dt}=\frac{dR_{Mg}}{dR_{set}}\cdot\frac{dR_{set}}{dt}=\frac{dR_{Mg}}{dR_{set}}\cdot\frac{dR_{set}}{df_{OSC}}\cdot\frac{df_{OSC}}{dt}$ | ( 6 ) |
| --- | --- | --- |

The set resistance used in this work is given by:

|  | $R_{set}=\frac{R_{fix}(R_{fix}+R_{Mg})}{2R_{fix}+R_{Mg}}$ | ( 7 ) |
| --- | --- | --- |

thus, the Mg resistance is:

|  | $R_{Mg}=\frac{2R_{set}R_{fix}-R_{fix}^{2}}{R_{fix}-R_{set}}$ | ( 8 ) |
| --- | --- | --- |

from which:

|  | $\frac{dR_{Mg}}{dR_{set}}=\frac{R_{fix}^{2}}{\left( R_{fix}-R_{set} \right)^{2}}$ | ( 9 ) |
| --- | --- | --- |

For the oscillator adopted in this work, it holds

|  | $f_{OSC}=\frac{\psi\alpha}{R_{set}}$ | ( 10 ) |
| --- | --- | --- |

where $\psi=10k\Omega\cdot10MHz$ and $\alpha=1;2$, hence:

|  | $\frac{dR_{Mg}}{dt}=-\frac{R_{fix}^{2}}{\left( R_{fix}-\frac{\psi\alpha}{f_{OSC}} \right)^{2}}\cdot\frac{\psi\alpha}{f_{OSC}^{2}}\cdot\frac{df_{OSC}}{dt}$ | ( 11 ) |
| --- | --- | --- |
|  | $R_{Mg}=\frac{R_{fix}(2\psi\alpha-R_{fix}f_{OSC})}{R_{fix}f_{OSC}-\psi\alpha}$ | ( 12 ) |

Thus, from ( 5 ), ( 11 ), ( 12 ), it is possible to derive the following expression:

|  | $WTR=-\frac{K\cdot\psi\cdot\alpha}{\left( 2\psi\alpha-R_{fix}f_{OSC} \right)^{2}}\cdot\frac{df_{OSC}}{dt}$ | ( 13 ) |
| --- | --- | --- |

This expression provides the operative equation to determine the WTR of the barrier as a function only of $f_{OSC}$ and ${df_{OSC}}/{dt}$ which are measured with the system proposed in this work.

**Correlation between WTR and wireless signal: generalized analytical model.** The analytical model can be generalized for a generic oscillator, whose oscillation frequency depends on the Mg resistance according to a set configuration. If $\mathcal{g}$ is the generic function that expresses this dependence, it holds:

|  | $f_{OSC}\mathcal{=G}\left( R_{Mg} \right)\Leftrightarrow R_{Mg}=\mathcal{G}^{-1}(f_{OSC})$ | ( 14 ) |
| --- | --- | --- |

Hence, the ( 5 ) can be written as follows:

|  | $WTR=\frac{K}{R_{Mg}^{2}}\cdot\frac{dR_{Mg}}{dt}=\frac{K}{\left[ \mathcal{G}^{-1}\left( f_{OSC} \right) \right]^{2}}\cdot\left[ \dot{\mathcal{G}^{-1}}\left( f_{OSC} \right) \right]$ | ( 15 ) |
| --- | --- | --- |

In more details, the proposed system can be devised with different types of oscillators and a different configuration of resistances, thus a general equation can be adopted and here a derivation is proposed. Let it be:

1. Constitutive equation of the oscillator:

|  | $f_{OSC}\mathcal{=F}\left( R_{set} \right)\Rightarrow\frac{df_{OSC}}{dR_{set}}\mathcal{=L (}known)$ | ( 16 ) |
| --- | --- | --- |

1. Configuration of resistances in the system:

|  | $R_{set}\mathcal{=H}\left( R_{i}, R_{Mg} \right)\Rightarrow\frac{dR_{set}}{dR_{Mg}}\mathcal{=J} (known)$ | ( 17 ) |
| --- | --- | --- |

where $i=1,\ldots,n$ with n the number of fixed resistances used in the system.

Hence,

|  | $\frac{dR_{Mg}}{dt}=\mathcal{J}^{-1}\cdot\mathcal{L}^{-1}\cdot\frac{df_{OSC}}{dt}$ | ( 18 ) |
| --- | --- | --- |

Therefore,

|  | $WTR=\frac{K}{R_{Mg}^{2}}\cdot\mathcal{J}^{-1}\cdot\mathcal{L}^{-1}\cdot\frac{df_{OSC}}{dt}=K\mathcal{\cdot X\cdot}\frac{df_{OSC}}{dt}$ | ( 19 ) |
| --- | --- | --- |

where $\mathcal{X=X}\left( f_{OSC} \right)={[\mathcal{L}^{-1}\cdot\mathcal{J}^{-1}]}/{R_{Mg}}$.

**Theory of water reactive diffusion in corroding Mg.** When the Mg is encapsulated by a barrier coating, water permeation is ruled by the water transport through the barrier and the water consumption in the Mg corrosion reaction. Thus, two governing equations should be considered: (i) diffusion equation for the water transport through the barrier and (ii) reactive diffusion equation for the water transport through metal with corrosion first-order kinetics. Hence, under the assumption of no side effects occurring if the thickness of the barrier is much smaller than the other dimensions, the diffusion occurs along the thickness direction (z-axis), so it holds:

|  | $\left\{ \begin{aligned} D_{B}\frac{\partial^{2}c}{\partial z^{2}}=\frac{\partial c}{\partial t} , h_{Mg}\leq z\leq h_{Mg}+h_{B} \\ D_{Mg}\frac{\partial^{2}c}{\partial z^{2}}-k_{Mg}c=\frac{\partial c}{\partial t}, 0\leq z\leq h_{Mg} \end{aligned} \right.$ | ( 20 ) |
| --- | --- | --- |

where $D_{B},D_{Mg}$ are the water diffusion coefficients in the barrier and in the Mg; $h_{Mg},h_{B}$ are the thicknesses of the Mg layer and the barrier encapsulation. The diffusion coefficients are assumed to be concentration-independent, according to the Fickian behavior.

The boundary conditions for the system of equations ( 20 ) are the following:

|  | $c\left( 0,t \right)=0, c\left( z,0 \right)=0, c\left( h_{Mg}+h_{B},t \right)=c_{0}$  $\left. \frac{\partial c}{\partial x} \right\vert_{x=0}=0, c\left( h_{Mg}^{-},t \right)=c\left( h_{Mg}^{+},t \right), D_{Mg}\left. \frac{\partial c}{\partial x} \right\vert_{x=h_{Mg}^{-}}=D_{B}\left. \frac{\partial c}{\partial x} \right\vert_{x=h_{Mg}^{+}}, (*)$ | ( 21 ) |
| --- | --- | --- |

where $h_{Mg}^{-},h_{Mg}^{+}$ represents the coordinates at the Mg/barrier interface inside the Mg layer and the barrier, respectively.

The solution of the system ( 20 ) is then given by:

|  | $c\left( z,t \right)=c_{0}\cdot\sum_{n=1}^{\infty} C_{n}e^{-\lambda_{n}t}\mathcal{L}_{n}\left( z \right)\mathcal{+M(}z)$ | ( 22 ) |
| --- | --- | --- |

with

|  | | $\mathcal{L}_{n}\left( z \right)=\left\{ \begin{aligned} \sin\left( \sqrt{\frac{\lambda_{n}}{D_{B}}}h_{B} \right)\cos\left( \sqrt{\frac{\lambda_{n}-k_{Mg}}{D_{Mg}}}z \right), 0\leq z\leq h_{Mg} \\ \cos\left( \sqrt{\frac{\lambda_{n}-k_{Mg}}{D_{Mg}}}h_{Mg} \right)\sin\left( \sqrt{\frac{\lambda_{n}}{D_{B}}}\left( h_{Mg}+h_{B}-z \right) \right), h\leq z\leq h_{Mg}+h_{B} \end{aligned} \right.$ | ( 23 ) | |  |
| --- | --- | --- | --- | --- | --- |
|  | | $\mathcal{M}\left( z \right)=\left\{ \begin{aligned} \alpha\cosh\left( \sqrt{\frac{k_{Mg}}{D_{Mg}}}z \right), 0\leq z\leq h_{Mg} \\ 1-\beta\left( h_{Mg}+h_{B}-z \right), h_{Mg}\leq z\leq h_{Mg}+h_{B} \end{aligned} \right.$ | ( 24 ) | |  |
|  | | $\alpha=\frac{1}{\sqrt{D_{Mg}k_{Mg}}\frac{h_{B}}{D_{B}}\sinh\theta+\cosh\theta}$ | ( 25 ) | |  |
|  | | $\beta=\frac{1}{\frac{D_{B}}{\sqrt{D_{Mg}k_{Mg}}}\coth\theta+h_{B}}$ | ( 26 ) | |  |
|  | | $\lambda_{n} eigenvalues of (*): \tan\sqrt{\frac{\lambda-k_{Mg}}{D_{Mg}}h_{Mg}^{2}}\tan\sqrt{\frac{\lambda}{D}h_{B}^{2}}=\sqrt{\frac{\lambda D_{B}}{\left( \lambda-k_{Mg} \right)D_{Mg}}}$ | ( 27 ) | |  |
|  | $C_{n}=\frac{-\frac{2}{\lambda_{n}}\sqrt{\lambda_{n}D_{B}}\cos\left( \sqrt{\frac{\lambda_{n}-k_{Mg}}{D_{Mg}}}h_{Mg} \right)}{h_{Mg}\sin^{2} \left( \sqrt{\frac{\lambda_{n}}{D}}h_{B} \right)\left[ 1+\frac{\sin\left( 2\sqrt{\frac{\lambda_{n}-k_{Mg}}{D_{Mg}}}h_{Mg} \right)}{2\sqrt{\frac{\lambda_{n}-k_{Mg}}{D_{Mg}}}h_{Mg}} \right]+h_{B}\cos^{2} \left( \sqrt{\frac{\lambda_{n}-k_{Mg}}{D_{Mg}}}h_{Mg} \right)\left[ 1-\frac{\sin\left( 2\sqrt{\frac{\lambda_{n}}{D_{B}}}h_{B} \right)}{2\sqrt{\frac{\lambda_{n}}{D_{B}}}h_{B}} \right]}$ | | | ( 28 ) | |

where $\theta^{2}={(k_{Mg}h_{Mg}^{2})}/{D_{Mg}}$ is the Thiele number, measuring the relative contributions between the reaction and the water transport.

The amount (thickness) of Mg corroded can be expressed by integration as follows:

| $\Delta h\left( t \right)=\frac{c_{0}M_{Mg}k_{Mg}h_{Mg}}{2\delta M_{H_{2}O}}\left[ \beta t\frac{\sinh\theta}{\theta} \right]+\sum_{n=1}^{\infty} \frac{C_{n}}{\lambda_{n}}\left( 1-e^{-\lambda_{n}t} \right)\frac{\sin\left( \sqrt{\frac{\lambda_{n}-k_{Mg}}{D_{Mg}}}h_{Mg} \right)}{\sqrt{\frac{\lambda_{n}-k_{Mg}}{D_{Mg}}}h_{Mg}}\sin\left( \sqrt{\frac{\lambda_{n}}{D_{B}}}h_{B} \right)$ | ( 29 ) |
| --- | --- |

which can be simplified to the following:

|  | $\Delta h\left( t \right)\approx\frac{c_{0}M_{Mg}}{2\delta M_{H_{2}O}}k_{Mg}h_{Mg}\beta\frac{\sinh\theta}{\theta}t=h_{Mg}\frac{t}{t_{c}^{B}}$ | ( 30 ) |
| --- | --- | --- |

where the lag for the completion of Mg corrosion is:

|  | $t_{c}^{B}=\frac{\frac{2\delta M_{H_{2}O}}{c_{0}M_{Mg}}h_{Mg}}{\sqrt{k_{Mg}D_{Mg}}\tanh\theta}\left( 1+\frac{h_{B}}{D_{B}}\sqrt{k_{Mg}D_{Mg}}\tanh\theta\right)$ | ( 31 ) |
| --- | --- | --- |

Noteworthy, $t_{c}^{B}$ increases with increasing Mg thickness as well as with increasing encapsulation thickness $h_{B}$ or with decreasing encapsulation’s diffusivity $D_{B}$, as expected. The described model represents a good theoretical framework for the experiments shown in the manuscript, but it could be further optimized by taking into account the barrier contribution of the hydroxide layer that gradually forms on top of Mg.

**Theory of permeability in multilayer laminates.** In case of a multi-layer barrier encapsulation, water permeation is governed by the same equation system ( 20 ) but the diffusion in the encapsulation depends on the specific single layers. In particular, the overall diffusion coefficient will depend on the diffusion coefficients of the single organic layers, on the defect distribution in the inorganic layers, on the total number of layers and on the their sequence [8,9]. Additionally, for a multi-layer system of organic encapsulations, it is possible to show that the overall water transmission rate is given by:

|  | $\frac{1}{WTR}=\sum_{k=1}^{N} \frac{1}{WTR_{i}}$ | ( 32 ) |
| --- | --- | --- |

where $WTR_{i}$ is the water transmission rate of the ith layer and $N$ is the total number of layers.

**Temperature-dependence of WTR and Arrhenius’ behavior.** The WTR of the barrier encapsulation, as well as its diffusion coefficient, solubility coefficient or permeability coefficient, is temperature-dependent and its behavior is governed by the Arrhenius law:

|  | $WTR=WTR_{0}e^{-\frac{E_{A}}{RT}}$ | ( 33 ) |
| --- | --- | --- |

where $E_{A} (J\cdot mol^{-1})$ is the activation energy for water diffusion in the barrier, $R=8.31 JK^{-1}mol^{-1}$ is the constant of perfect gases, $T$ is the absolute temperature (K), $WTR_{0}$ is a constant parameter.

Thus, the following relation holds for two distinct temperatures:

|  | $WTR_{2}=WTR_{1}e^{-\frac{E_{A}}{R}\left( \frac{1}{T_{2}}-\frac{1}{T_{1}} \right)}$ | ( 34 ) |
| --- | --- | --- |

where $WTR_{1},WTR_{2}$ are the WTR values at temperatures $T_{1},T_{2}$, respectively.

In polymers, the activation energy $E_{A}$ changes when the temperature is increased above the glass transition ($T_{g}$), which in turn depends on the amount of absorbed water.

**Model of Mg corrosion that accounts for defects in the encapsulation.** The previous model of Mg corrosion considers the encapsulation as defect-free with homogeneous water diffusion. However, in reality encapsulations can present a certain density of pristine defects that may result from the deposition process and environmental conditions. These defects, especially pinholes within inorganic layers, edge defects or defects at the organic/inorganic and Mg/encapsulation interfaces provide pathways for rapid water permeation and localised Mg corrosion (Figure S1(a)). The influence of the defect population is therefore integrated into the temporal variation of the resistance of the Mg sensor from which the WTR is calculated. This means that the WTR, acquired experimentally as proportional to the temporal variation of the Mg resistance, is averaged over the film as a whole, including the defects, and results in a higher value compared to the WTR of the defect-free encapsulation.

Although there are many previous examples of models to account for the effect of local flaws in polymeric encapsulations [10,11], we propose here a simplified model that is adapted to the Mg electrical test.

Let us consider an encapsulation with a given fraction of defective area ($\Delta$):

|  | $\Delta=\frac{S^{D}}{S}$ | ( 35 ) |
| --- | --- | --- |

where $S^{D},S$ are the total defects’ surface and the total surface of the encapsulation.

Below each defect in the encapsulation, there will be a corroded area (called hereafter “spot”) in the Mg film with rapid water permeation. We assume that under each defect, the spot spreads through the thickness of the Mg film. As sketched in Figure S1(a), the Mg film area is divided into spot-free areas and spot-containing areas, each of width $w$ and perpendicular to the axis of resistance measurement, with isolated, non-overlapping circular spots of radius $r$. Each spot-containing area (width $w$, length $2r$) is split by each spot in two regions, whose electrical resistances are connected in parallel (assuming that the spot is empty of Mg). Thus, on a first approximation the conductance of one spot-containing Mg area is given by:

|  | $G^{D}=\frac{\left( w-2r \right)h}{2\rho r}$ | ( 36 ) |
| --- | --- | --- |

where$\rho$ is the Mg electrical resistivity and $h$ is the Mg thickness and $w$ is the total width of the Mg film area (Figure S1(a)).

The resistance of all the spot-containing Mg areas is:

|  | $R_{T}^{D}=\frac{2\rho r\Delta}{\left( w-2r \right)h}$ | ( 37 ) |
| --- | --- | --- |

The resistance of all the spot-free Mg areas is:

|  | $R_{T}^{DF}=\frac{\rho(L-2r\Delta)}{wh}$ | ( 38 ) |
| --- | --- | --- |

Thus, the total Mg resistance and conductance are:

|  | $R_{Mg}=R_{T}^{D}+R_{T}^{DF}=\frac{\rho}{h}\underset{\xi}{\underbrace{\left( \frac{L}{h}+\frac{{4r}^{2}\Delta}{w(w-2r)} \right)}}=\frac{\rho\xi}{h}$ | ( 39 ) |
| --- | --- | --- |
|  | $G_{Mg}=\frac{h}{\rho\xi}$ | ( 40 ) |

From the Mg corrosion equation, one can show that the WTR of the encapsulation is given by:

|  | $WTR=-2\left( \frac{M_{H_{2}O}}{M_{Mg}} \right)\frac{\delta Lw}{S}\rho\xi\frac{dG_{Mg}}{dt}$ | ( 41 ) |
| --- | --- | --- |

where $\delta$ is the Mg mass density; $M_{H_{2}O},M_{Mg}$ are the molecular weights of water and Mg, respectively.

We can write:

|  | $WTR=\underset{K}{\underbrace{-2\left( \frac{M_{H_{2}O}}{M_{Mg}} \right)\left( \frac{S_{Mg}}{S} \right)\delta\rho\frac{L}{w}}}\cdot\frac{dG_{Mg}}{dt}\underset{K^{D}}{\underbrace{-2\left( \frac{M_{H_{2}O}}{M_{Mg}} \right) \delta\rho\frac{4r^{2}}{w\left( w-2r \right)}\frac{\Delta}{1-\Delta}}}\cdot\frac{dG_{Mg}}{dt}$ | ( 42 ) |
| --- | --- | --- |
|  | $WTR=K\frac{dG_{Mg}}{dt}+K^{D}\frac{dG_{Mg}}{dt}=WTR_{DF}+WTR_{D}$ | ( 43 ) |

where $K,K^{D}$ are two constants that only depend on physical constants, specimen geometry and defect density, and $WTR_{DF},WTR_{D}$ are the water transmission rates for the defect-free encapsulation and for the defects, respectively. By comparing the ( 5 ) and ( 43 ), it can be deduced that the proposed experimental method provides the WTR of the defect-free encapsulation ($WTR_{DF}=K{dG_{Mg}}/{dt}$): to obtain the value of the WTR corresponding to the encapsulation with pre-existing defects, the term $WTR_{D}=K^{D}{dG_{Mg}}/{dt}$, should be added. The fraction of defective area $\Delta$ can be quantified by optical observation and image processing (before implantation), and also the defect size ($r$) can be obtained with standard microscopy techniques (e.g. SEM, AFM); thus, $K^{D}$ can be calculated and the WTR for the defective encapsulation can be extracted.

As an example, considering a defect size of ~100 µm^2^ ($r\approx5\mu m$) in polyimide films with defect density of 0.2 cm^-2^ [12] (Figure S1(b)), then $\Delta=2\times{10}^{-7}$, and (with $w=100 \mu m$) $K^{D}=5.35\times{10}^{-14} g\Omega m^{-2}$. The low value of this coefficient compared to $K$ ($\sim{10}^{-4}g\Omega m^{-2}$) confirms that for polymers such as polyimide, the contribution of defects ($WTR_{D}$) is minimal compared to the diffusive mechanism ($WTR_{DF}$).

In the case of more defective encapsulations, e.g. polymers coated with inorganic layers or hybrid organic-inorganic multilayers, the size and density of defects can lead to a remarkable deviation from the defect-free WTR ($WTR_{DF}$). In fact, the fraction $\Delta$ can be higher, due to the presence of multiple inorganic layers, therefore, higher orders of magnitude for $K^{D}$ can be achieved. Figure S1(c) illustrates the variation of $K^{D}$ as a function of $\Delta$ and $r$ (the latter in the range of 0-10 µm and in 0-1µm), for different values of the width $w$ of the Mg film area. As expected, $K^{D}$ increases both with $\Delta$ and $r$, and decreases with $w$, reaching peaks of ~$3\times{10}^{-4},\sim5\times{10}^{-6}$, ~$4\times{10}^{-8}$ (with $\Delta={10}^{-3},r=10\mu m$) for $w=25\mu m, 100\mu m, 1000\mu m$, respectively. The value of $WTR$ can thus deviate more or less remarkably from $WTR_{DF}$, depending on the order of magnitude of $K^{D}$. Considering for instance, a defect density of ~100 cm^-2^ for an ALD Al_2_O_3_ layer [13], then $\Delta={10}^{-4}$ and $K^{D}\approx{5\times10}^{-9}g\Omega m^{-2}$ (with $w=100 \mu m, r=200 nm$). This value is noticeably higher than that for a standard polymer, but it is still negligible if compared with the order of magnitude of $WTR_{DF}$.

The density of defects can also be extracted by measuring the apparent WTR of the unencapsulated Mg: this transmission rate is the same as $WTR_{D}$, since in correspondence to the defects of the encapsulation, Mg is not coated.


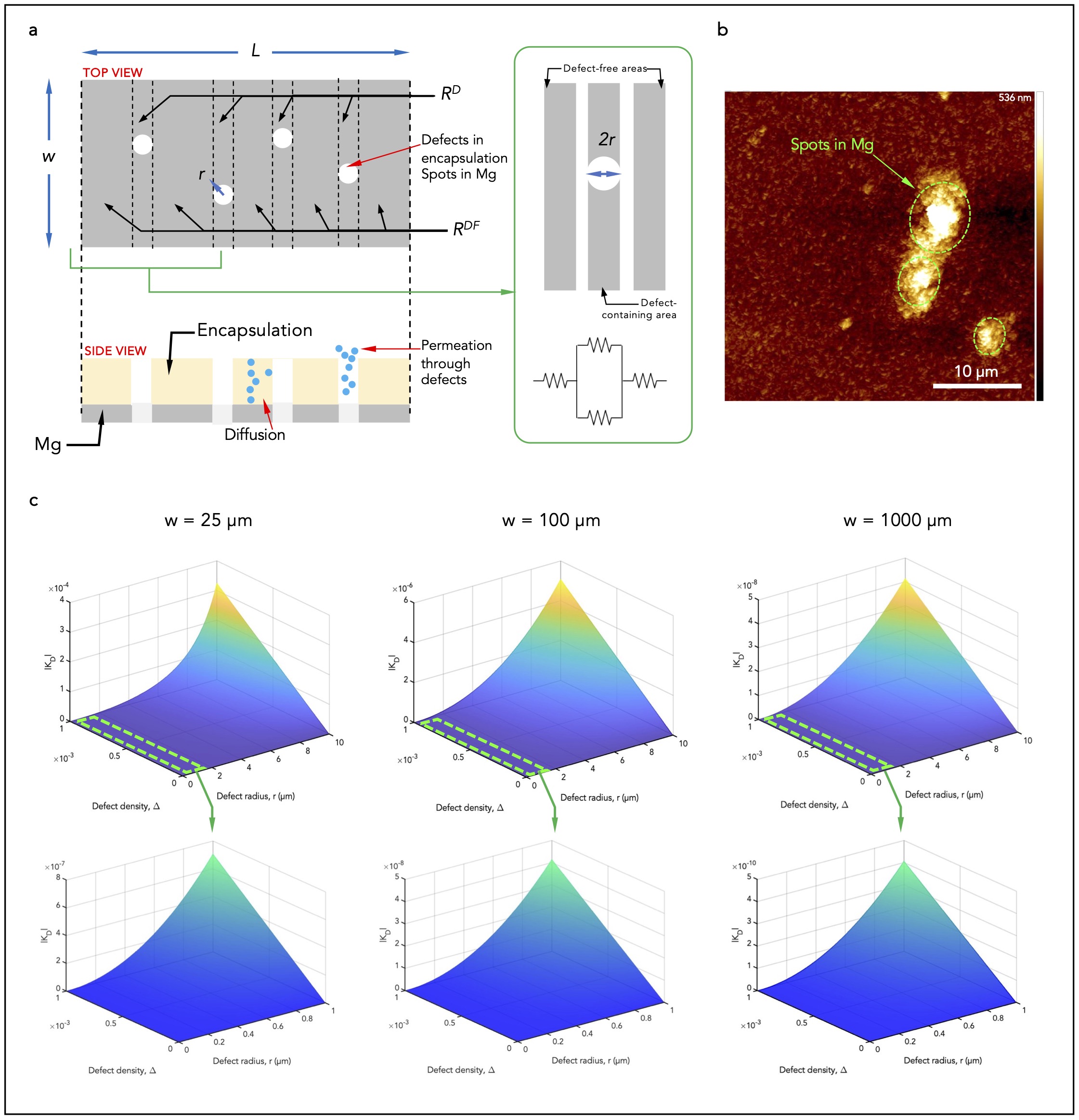


**Figure S1.** (a) Scheme of defects present in the thin-film encapsulation coating the Mg film. (b) AFM topography image showing three corrosion spots in the Mg film due to the presence of pinhole defects in the encapsulation (removed before the imaging). (c) 3D plots of K_D_ (units $g\Omega m^{-2}$) as function of the defect radius and the defect density (fraction $\Delta$), for different widths of Mg tracks (25, 100, 1000 µm).

Supplementary Note 1

Smith Chart


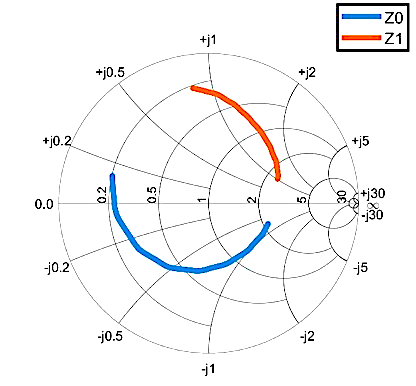


**Figure S2.** Smith Chart plot of impedances of the flexible antenna without (Z0) and with (Z1) matching circuit.

Supplementary Note 2

Fabrication of flexible Mg test sensors


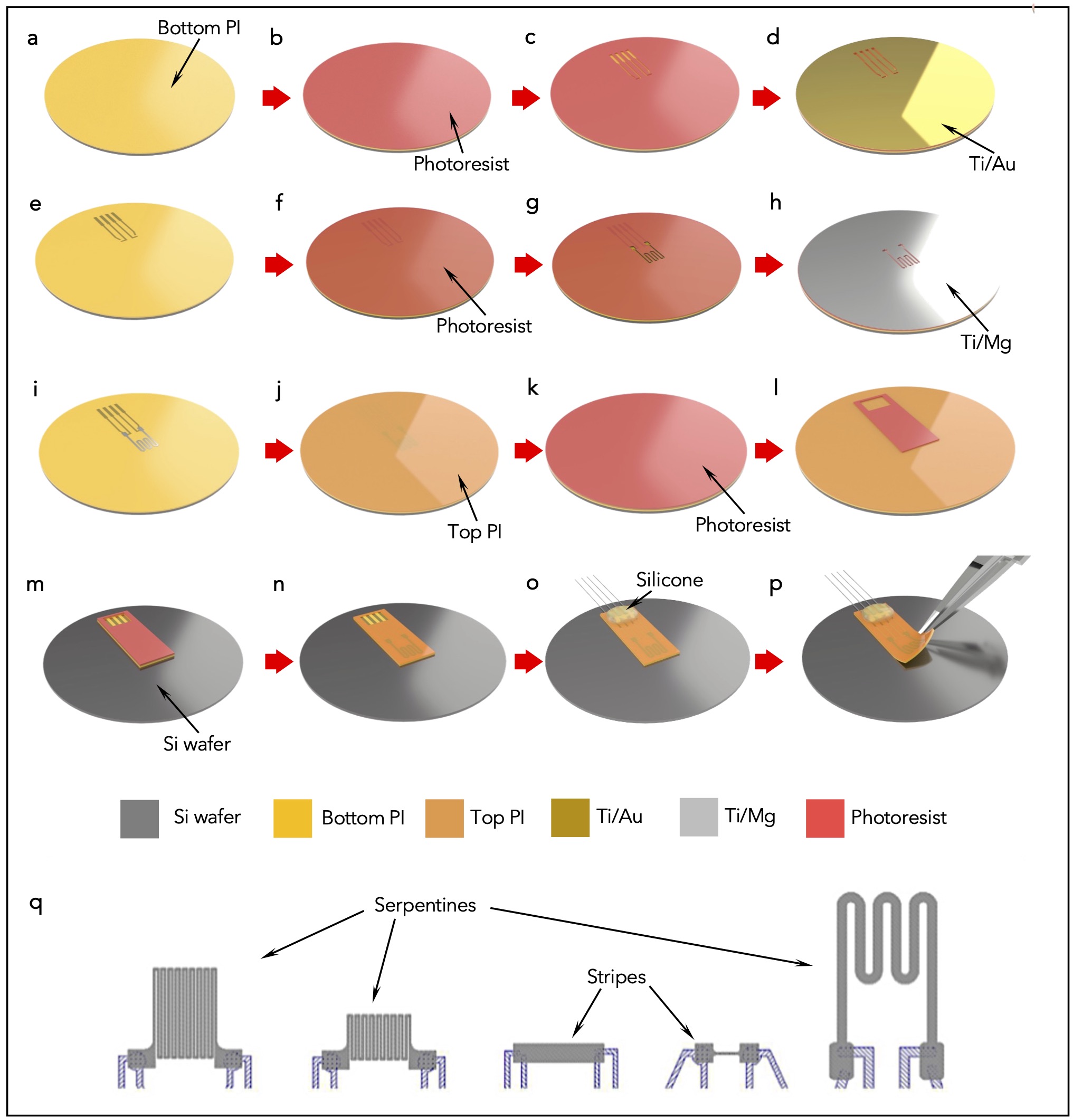


**Figure S3.** Fabrication process of the flexible Mg test sensors. (a) Spin-coating and curing of PI substrate. (b) Spin-coating and baking of photoresist. (c) First photolithography. (d) Reactive sputtering Ti/Pt interconnections. (e) First liftoff, (f) Spin-coating and baking of photoresist. (g) Second photolithography. (h) Reactive sputtering of Ti adhesion layer and thermal evaporation of Mg sensing layer. (i) Second liftoff. (j) Spin-coating and curing of the top PI substrate. (k) Spin-coating and baking of photoresist. (l) Third photolithography. (m) RIE dry etching of PI. (n) Resist stripping. (o) Wiring and immobilization of connections with silicone. (p) Peeling-off. (q) Designs selected for patterning the Mg test sensors.

Supplementary Note 3

Mg test sensors: mechanical properties

***
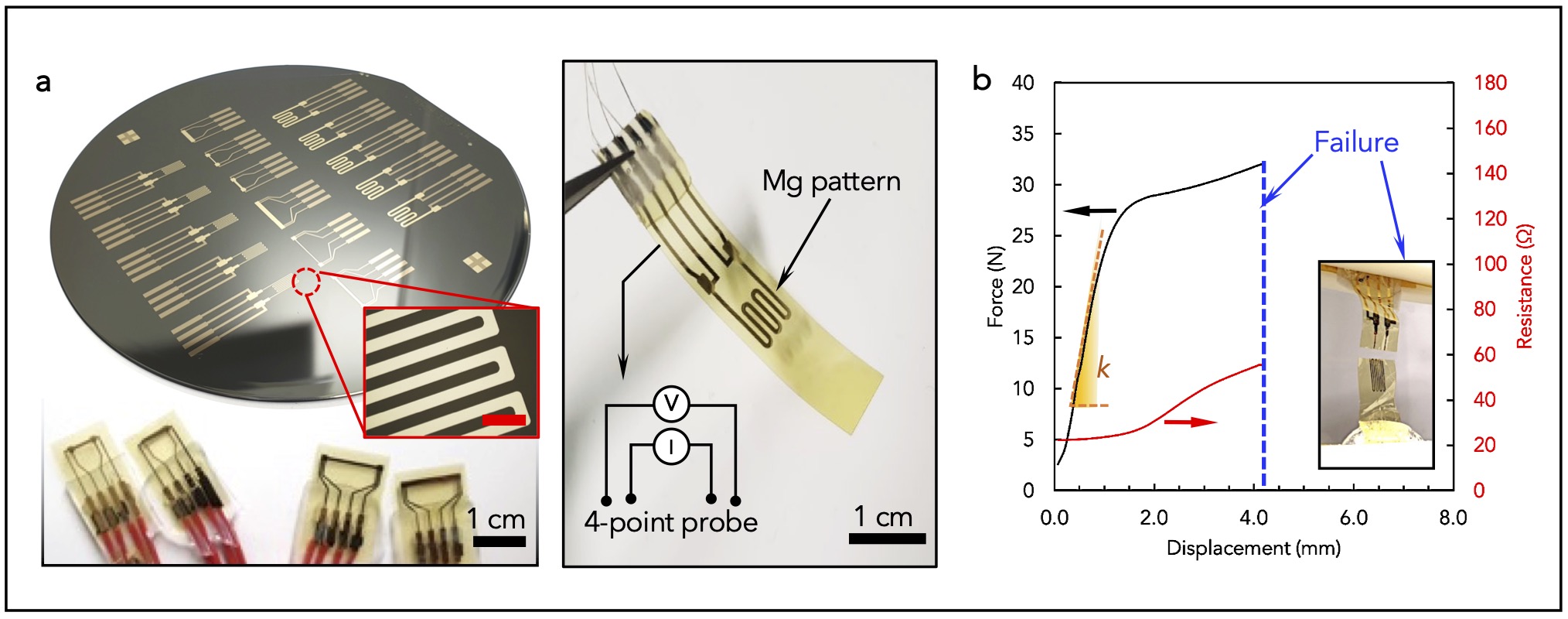
***

**Figure S4.** (a) Photographs of the fabricated devices with several different designs. Red scale bar: 200 µm. (b) Force-displacement and resistance-displacement curves of the Mg flexible test sensors undergoing mechanical tensile tests (with indication of the elastic constant k).

By applying the rule of mixtures to the PI/Ti/Mg/PI stacking sequence, the effective Young’s modulus of 21.08 GPa was obtained for the Ti/Mg bilayer. This is lower than the Young’s moduli of the bulk metals (106 GPa for Ti; 42-45 GPa for Mg): this can be ascribed to the effect of the deposition process onto the microstructure, in fact Mg thin films present hexagonal grains that are less interpenetrated than in the bulk counterparts, contributing in decreasing the stiffness. Gravimetric measurements yielded a Ti/Mg film density of (1.876 ± 0.197) gcm^-3^: given that ~90% of the total thickness is occupied by Mg, an approximated calculation gives a Mg mass density of 1.688 gcm^-3^, of the same order as the bulk-Mg mass density but slightly lower, which could also explain the lower stiffness.

Supplementary Note 4

Oscillation frequency and set resistance.


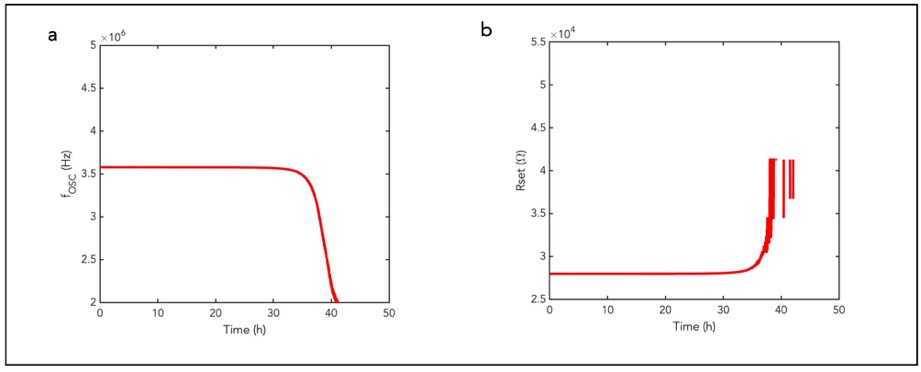


**Figure S5.** Temporal evolution of the oscillation frequency (a) and the set resistance (b).

Supplementary Note 5

**
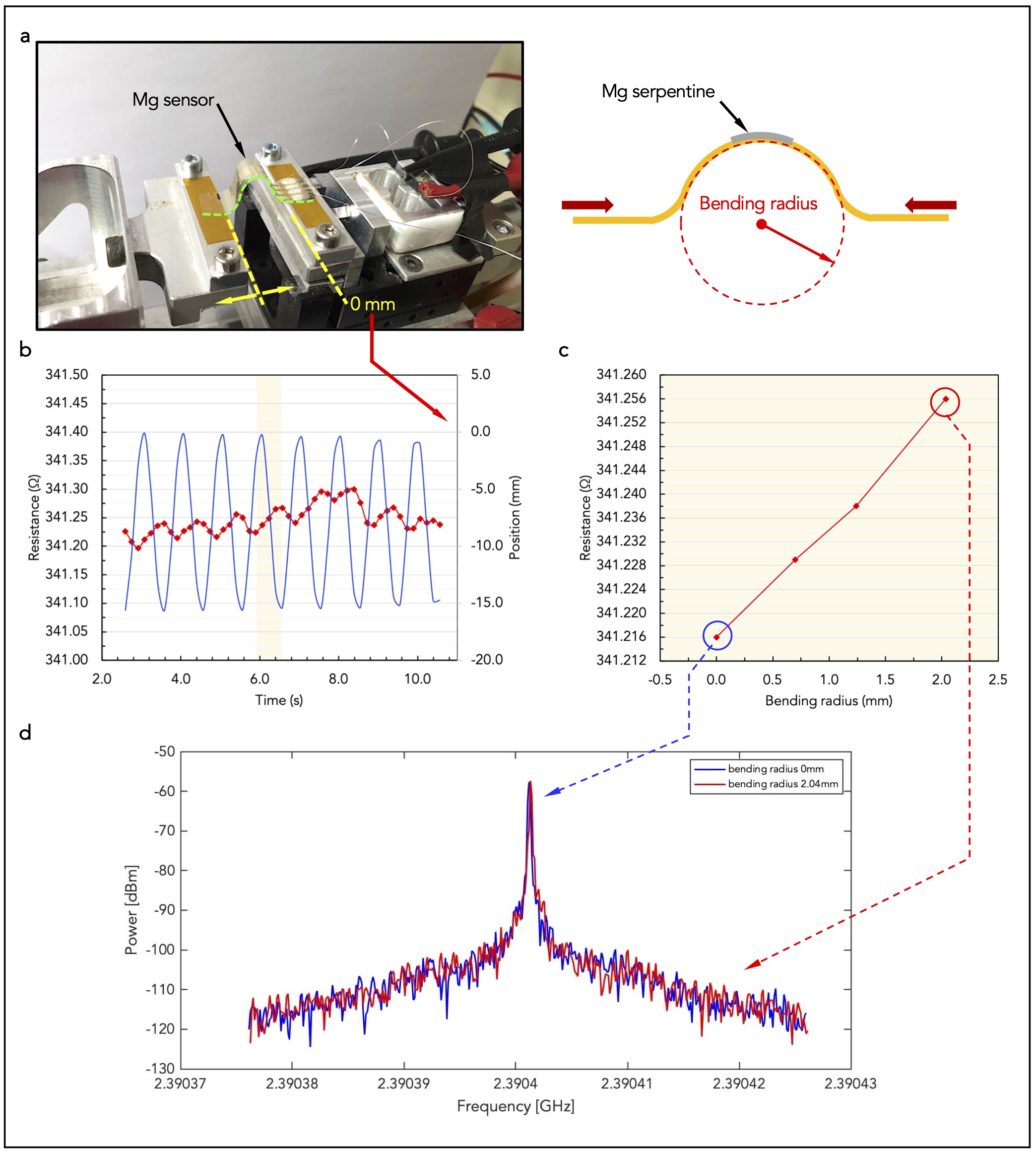
**

**Figure S6.** (a) Mechanical setup for cyclic buckling of Mg sensor. On the right the scheme shows the bending radius exhibited by the Mg sensor. (b) Cyclic buckling test (15 mm displacement, 1 Hz frequency) and simultaneous monitoring of Mg electrical resistance. (c) Dependence of Mg resistance on bending radius: the resistance increases within 0.1Ω. (d) Backscattered signals for a Mg sensor with two bending radii, i.e. 0mm and 2.04 mm. There is no detectable difference between the two signals: although the different Mg resistance induces a shift in the oscillation frequency, this shift is not detectable because very low compared to the value of the oscillation frequency.

Supplementary Note 6

WTR for different Mg designs


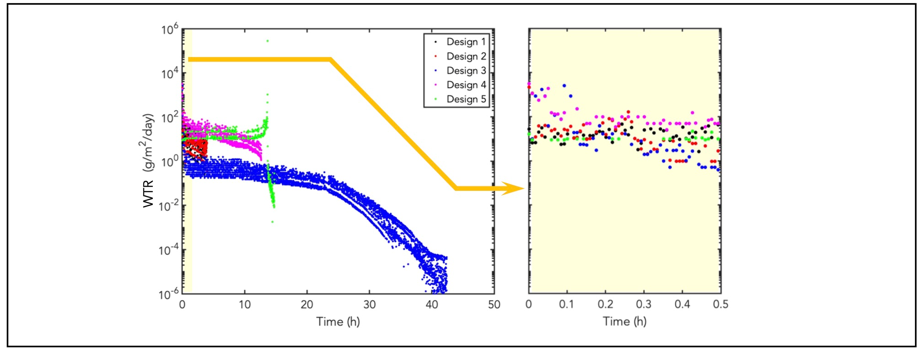


**Figure S7.** Comparison of the WVTR curves for different selected designs of the Mg test sensors.

Supplementary Note 7

Choice of oscillator


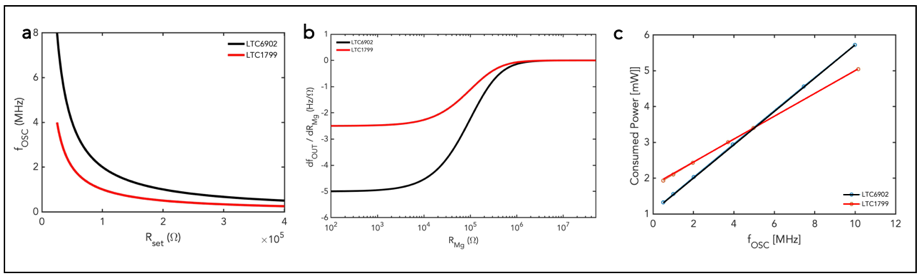


**Figure S8.** Performance comparison between two selected oscillators for the WPS circuitry. Curves of oscillation frequency vs Rset (a), dfout/dRMg vs RMg (b) and consumed power (c).

Supplementary Note 8

Hybrid organic-inorganic multilayer encapsulations


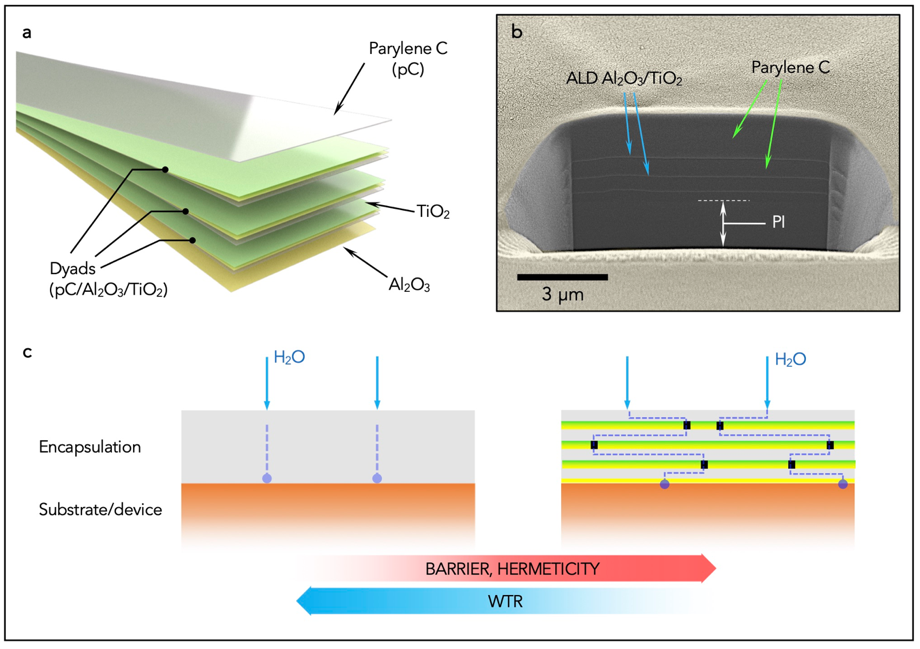


**Figure S9.** Illustration (a) and SEM image of FIB-milled cross section (b) of a 3-dyad Parylene C-Al_2_O_3_-TiO_2_ encapsulation. (c) Illustration of the concept of defect-decoupling and increased barrier for hybrid multilayer encapsulations; the black spots represent the cracks in the inorganic layers.

Supplementary Note 9

Defect-dominated water permeation


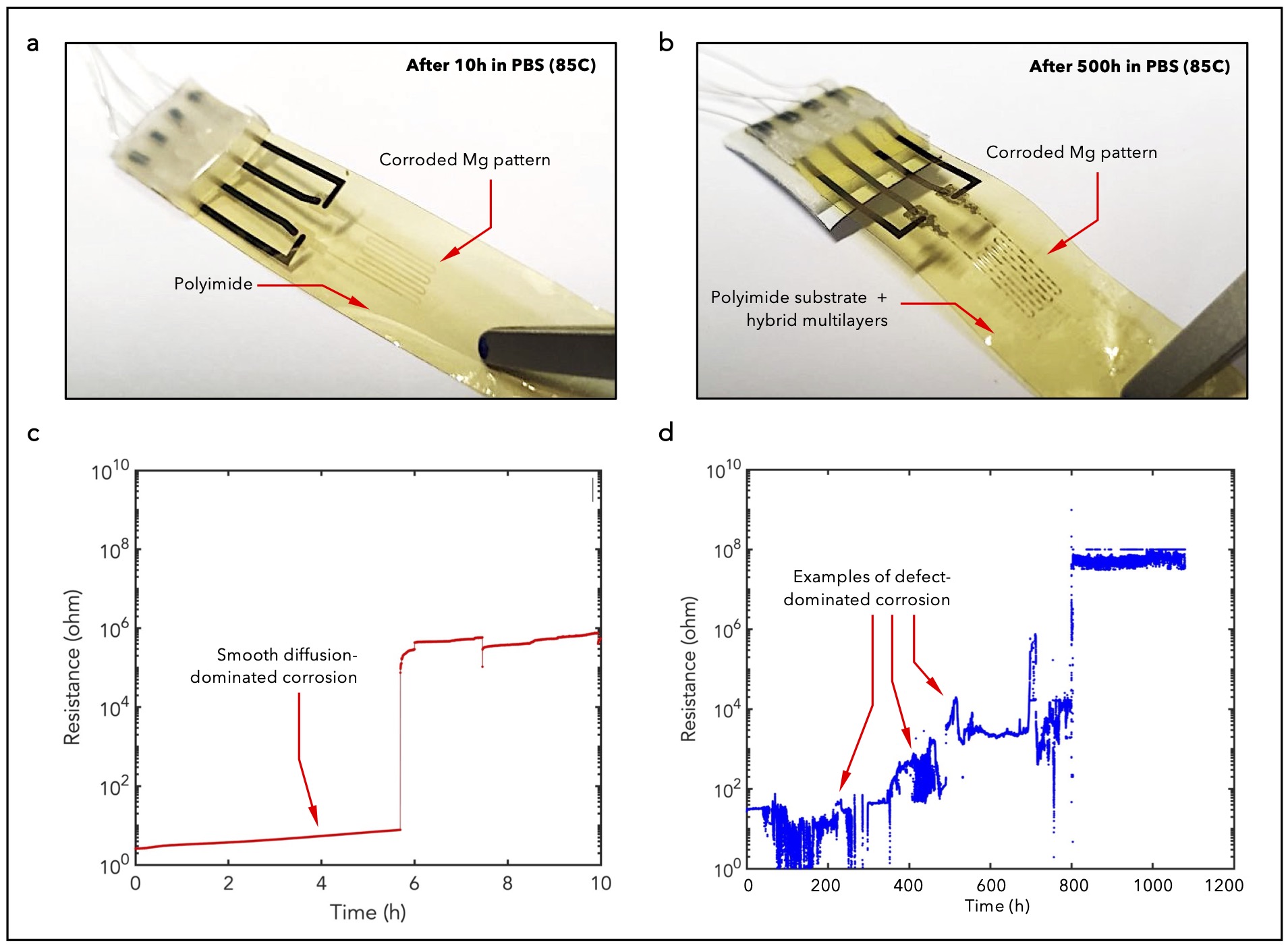


**Figure S10.** (a, b) Photos of Mg sensors soaked in PBS at 85°C, encapsulated with a 5 µu-thick polyimide film and removed from PBS after 10h (a), or encapsulated with a ~3µm-thick 3-dyad parylene C/Al_2_O_3_ multilayer on a polyimide substrate and removed from PBS after 500h (b). (c, d) Resistance curves for the two Mg sensors depicted in (a, b), respectively. The curve in (c) exhibits a smooth monotonic resistance increase due to diffusion-dominated corrosion; the curve in (d) exhibits a fragmented resistance increase due to defect-dominated corrosion.

Supplementary Note 10

Fabrication of the flexible i-WPS


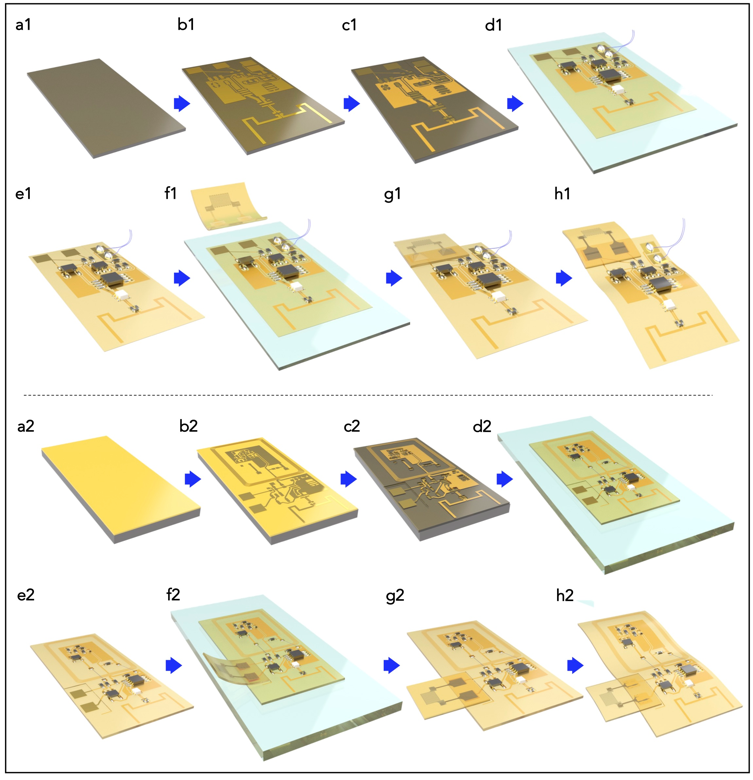


**Figure S11.** Fabrication and assembly steps of the flexible i-WPS: (a1-h1) externally-powered i-WPS, (a2-h2) remotely-powered i-WPS. (a1, a2) Spin-coating and curing of PI substrate. (b1, b2) Reactive sputtering of Ti/Au metallic interconnections and circuits. (c1, c2) RIE dry etching of PI to expose the connection pads. (d1, d2) Pick and place and soldering of the electrical components on the i-WPS peeled off and positioned on a glass substrate. (e1, e2) Deposition of the selected encapsulation. (f1, f2) Alignment and soldering of the disposable Mg insert. (g1, g2) Release and coating with PIB and silicone the electrical components. (h1, h2) Peeling-off of the final i-WPS.

Supplementary Note 11

Design of the flexible i-WPS


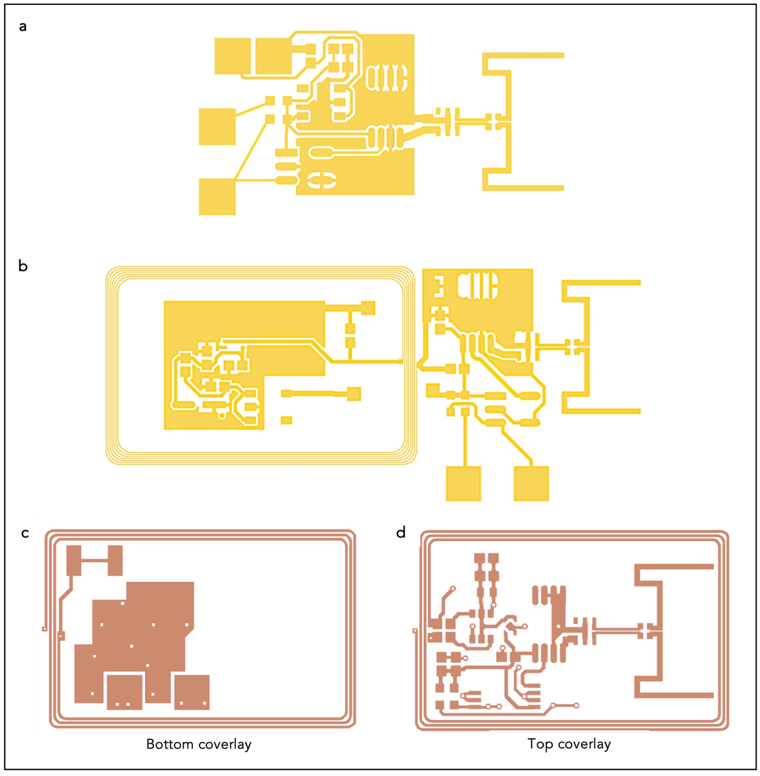


**Figure S12.** Designs of the (a) externally-powered i-WPS, (b)remotely-powered i-WPS, (c, d) miniaturized i-WPS.

Supplementary Note 12

Mg inserts, externally powered i-WPS, wireless antenna and powering coil


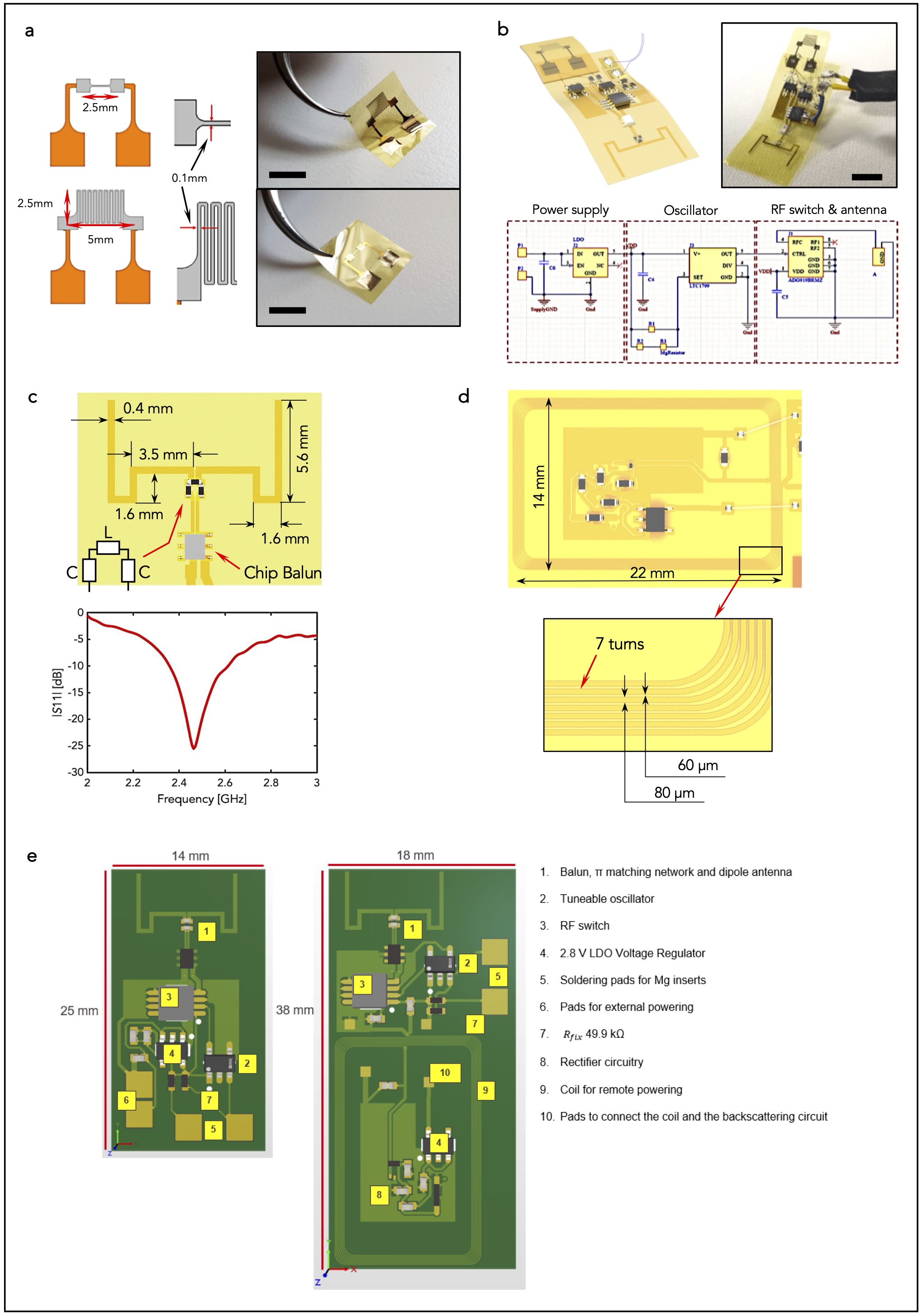


**Figure S13.** (a) Designs and photos of the Mg flexible inserts. Scale bar: 5 mm. (b) Illustration and circuit of the externally powered i-WPS. Scale bar: 5 mm. (c) Design of the flexible implantable antenna and plot of the corresponding S_11_ coefficient. (d) Design of the coil for the wireless powering unit. (e) Layout of the externally powered and remotely powered i-WPS.

Supplementary Note 13

Design of implantable antennas


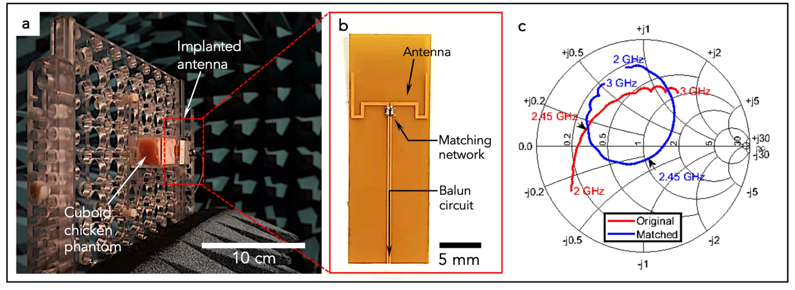


**Figure S14.** Measurements of the designed flexible implantable antenna used in the i-WPS. (a) Photo of the measurement platform in an electromagnetic anechoic chamber, where the antenna was implanted in a cuboid chicken phantom. (b) Photo of a flexible device with the designed antenna. (c) The input impedance of the original implanted antenna and the matched implanted antenna connected to the matching network shown in the Smith chart.

Supplementary Note 14

Subcutaneous implantation in a small rodent


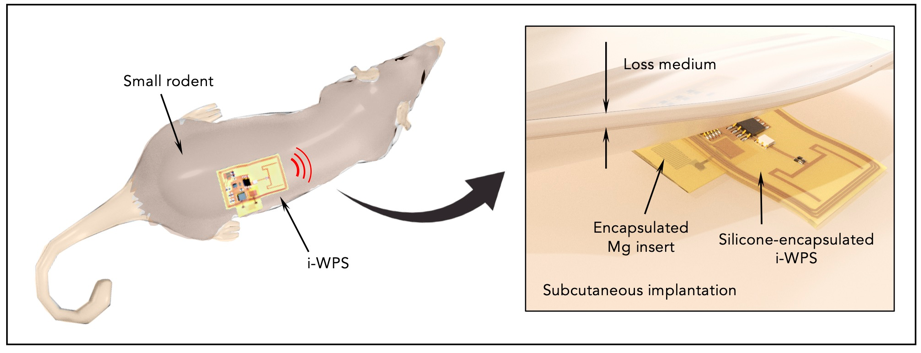


**Figure S15.** Illustration of the subcutaneous implantation of the miniaturized i-WPS in a small rodent (mouse, rat).

Supplementary Note 15

Definition of scattering parameter S_11_

The reflection coefficient in scattering parameters for the antenna measurement is defined as follows:

|  | $S_{11}[dB]=20\log_{10} \vert\Gamma\vert$ | ( 44 ) |
| --- | --- | --- |

in which$\Gamma$is the reflection coefficient of the voltage waves at port 1:

|  | $\Gamma=\left\vert\frac{b_{1}}{a_{1}} \right\vert$ | ( 45 ) |
| --- | --- | --- |

where $a_{1},b_{1}$ are the incident and reflected voltage waves at the antenna feed port. This parameter is directly measured through a vector network analyzer connected to the antenna under test in an electromagnetic anechoic chamber.

According to the definition of reflection coefficient, it can also be written in the form of characteristic impedance at the reference interface

|  | $\Gamma=\frac{Z_{i}-Z_{0}}{Z_{i}+Z_{0}}$ | ( 46 ) |
| --- | --- | --- |

where $Z_{0}$ is the characteristic impedance of the feed line/caxial cable, and $Z_{i}$ is the input impedance of the antenna. Similarly, in case of wireless powering between two coils via inductive coupling, the input impedance of the transmitter coil under test can be written as:

|  | $Z_{i}=\frac{V}{I}=Z_{1}+\frac{\omega^{2}L_{M}^{2}}{Z_{2}}$ | ( 47 ) |
| --- | --- | --- |

where $V$ is the input voltage; $I$ is the current flowing into the coil; $Z_{1}, Z_{2}$ are the self-impedances of the transmitter coil connected to a power source and the receiver coil (depending on the resistance, inductance and capacitance of each element); $\omega$ is the angular frequency of the driving source and $L_{M}$ is the mutual inductance coefficient. In the case of the proposed miniaturized WPS remotely powered through an external coil of wider area, $L_{M}$ can be considered negligible due to weak inductive coupling.


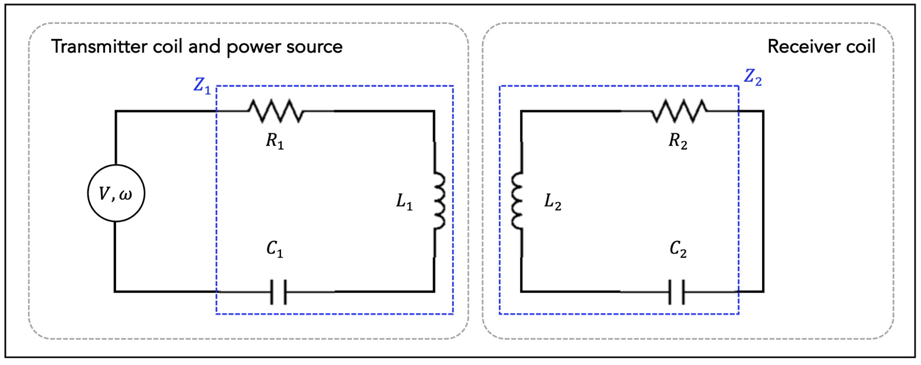


**Figure S16.** Wireless inductive coupling between the transmitter coil connected to a power source and the receiver coil.

Supplementary Note 16

Design of wireless powering unit


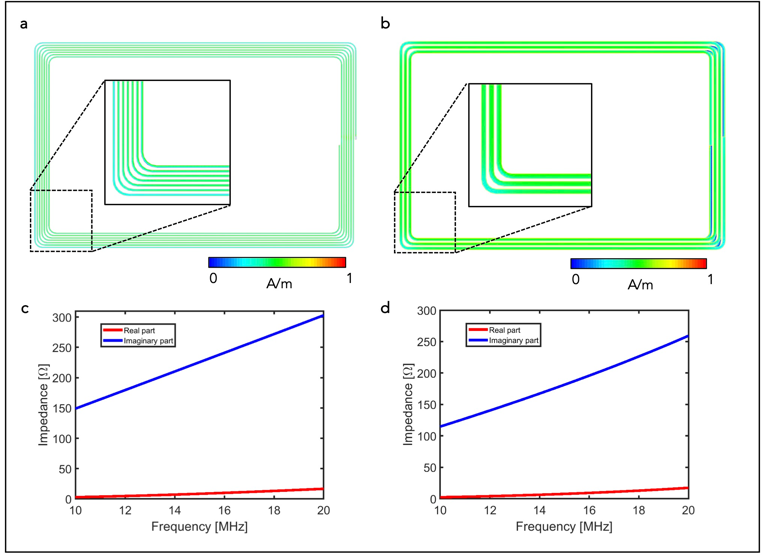


**Figure S17.** Full-wave electromagnetic simulations of the receiver coils on the wireless i-WPS using CST Studio Suite: (a, b) Surface current distribution (after normalization) over the metal coverlay of the coils used in wireless-remotely powered i-WPS (a) and miniaturized i-WPS (b). (c, d) Input impedance of the coils used in wireless-remotely powered i-WPS (c) and miniaturized i-WPS (d).

Supplementary Note 17

Miniaturized i-WPS


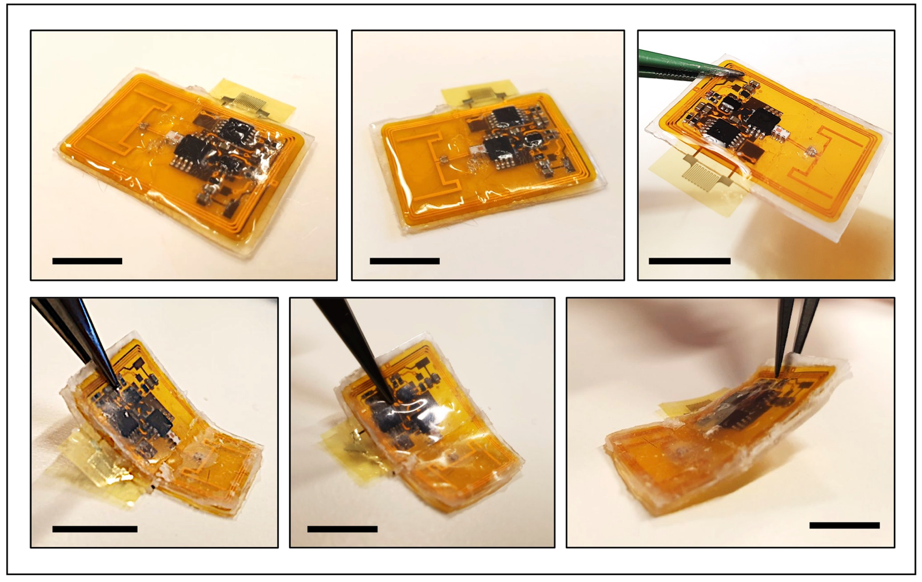


**Figure S18.** Photos of the miniaturized i-WPS. The silicone encapsulation provides softness and tissue-adaptability. Scale bar: 1 cm.


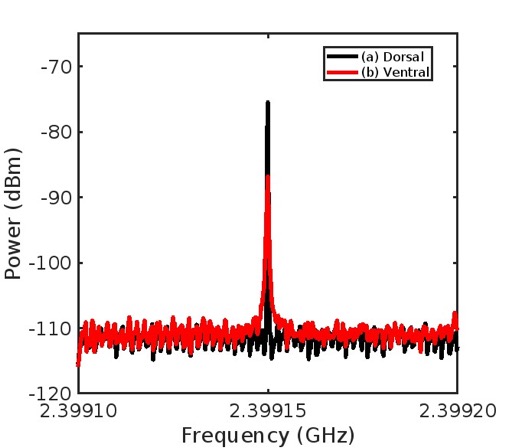


**Figure S19.** Backscattered signal detected by the mini-i-WPS implanted in different positions in the animal body: dorsal or back-up (a) and ventral or belly-up (b).

Supplementary Note 18

Contribution of bending, tilting, rotation of the animal body.


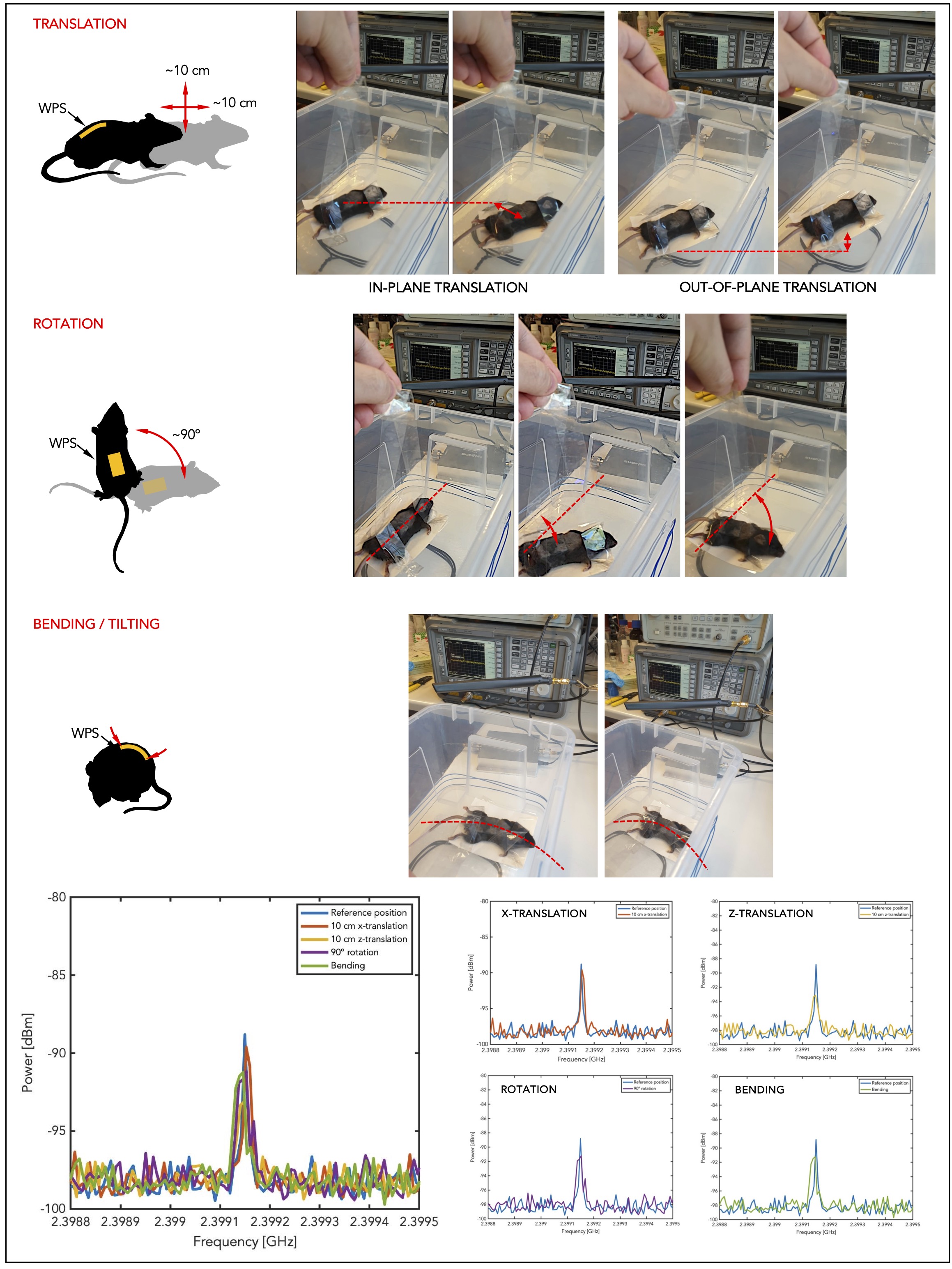


**Figure S20.** Backscattered signals detected by the mini-i-WPS implanted in the animal body subjected to different movements: in-plane translation, out-of-plane translation, rotation, bending. Although minimal variation in the signal amplitude can be observed, there is no variation in the oscillation frequency, providing a robust validation for the proposed wireless platform.

**Data availability:** All data supporting the findings of this study are available within the paper and its Supplementary Information. Source Data are provided with this paper.

References

1. Park, S.I.; Shin, G.; McCall, J.G.; Al-Hasani, R.; Norris, A.; Xia, L.; Brenner, D.S.; Noh, K.N.; Bang, S.Y.; Bhatti, D.L.; et al. Stretchable Multichannel Antennas in Soft Wireless Optoelectronic Implants for Optogenetics. *Proc. Natl. Acad. Sci.* **2016**, *113*, E8169–E8177, doi:10.1073/pnas.1611769113.

2. Skrivervik, A.; Bosiljevac, M.; Sipus, Z. Fundamental Limits for Implanted Antennas: Maximum Power Density Reaching Free Space. *IEEE Trans. Antennas Propag.* **2019**, *PP*, 1–1, doi:10.1109/TAP.2019.2891697.

3. Gao, M.; Sipus, Z.; Skrivervik, A.K. Analytic Approximation of In-Body Path Loss for Implanted Antennas. *IEEE Open J. Antennas Propag.* **2023**, *4*, 537–545, doi:10.1109/OJAP.2023.3276686.

4. Gao, M.; Rosenthal, J.D.; Wu, K.; Ramírez, G.A.; Šipuš, Z.; Lacour, S.P.; Skrivervik, A.K. Radiation Patterns of RF Wireless Devices Implanted in Small Animals: Unexpected Deformations Due to Body Resonance. *IEEE Trans. Biomed. Circuits Syst.* **2024**, *18*, 27–38, doi:10.1109/TBCAS.2023.3300649.

5. Shin, G.; Gomez, A.M.; Al-Hasani, R.; Jeong, Y.R.; Kim, J.; Xie, Z.; Banks, A.; Lee, S.M.; Han, S.Y.; Yoo, C.J.; et al. Flexible Near-Field Wireless Optoelectronics as Subdermal Implants for Broad Applications in Optogenetics. *Neuron* **2017**, *93*, 509-521.e3, doi:10.1016/j.neuron.2016.12.031.

6. Singer, A.; Robinson, J.T. Wireless Power Delivery Techniques for Miniature Implantable Bioelectronics. *Adv. Healthc. Mater.* **2021**, *10*, 2100664, doi:10.1002/adhm.202100664.

7. Mariello, M.; Ayama, K.; Wu, K.; Baudino, C.; Wang, L.; Mutschler, L.; Jourdan, L.A.; Cleusix, M.B.; Furfaro, I.; Kathe, C.; et al. Magnesium Test: Universal and Ultra-Sensitive Method for Measuring Reliability of Thin-Film-Encapsulated Bioelectronic Implants in Physiological Environment. *Adv. Funct. Mater.* **2024**, *n/a*, 2315420, doi:10.1002/adfm.202315420.

8. Graff, G.L.; Williford, R.E.; Burrows, P.E. Mechanisms of Vapor Permeation through Multilayer Barrier Films: Lag Time versus Equilibrium Permeation. *J. Appl. Phys.* **2004**, *96*, 1840–1849, doi:10.1063/1.1768610.

9. Langowski, H.-C. Permeation of Gases and Condensable Substances through Monolayer and Multilayer Structures. In *Plastic Packaging*; John Wiley & Sons, Ltd, 2008; pp. 297–347 ISBN 978-3-527-62142-2.

10. Rossi, G.; Nulman, M. Effect of Local Flaws in Polymeric Permeation Reducing Barriers. *J. Appl. Phys.* **1993**, *74*, 5471–5475, doi:10.1063/1.354227.

11. da Silva Sobrinho, A.S.; Czeremuszkin, G.; Latrèche, M.; Wertheimer, M.R. Defect-Permeation Correlation for Ultrathin Transparent Barrier Coatings on Polymers. *J. Vac. Sci. Technol. A* **2000**, *18*, 149–157, doi:10.1116/1.582156.

12. Rubehn, B.; Stieglitz, T. Measurement of Defects in Spin Coated Polyimide Films. In Proceedings of the 2007 29th Annual International Conference of the IEEE Engineering in Medicine and Biology Society; August 2007; pp. 183–185.

13. Zhang, Y.; Seghete, D.; Abdulagatov, A.; Gibbs, Z.; Cavanagh, A.; Yang, R.; George, S.; Lee, Y.-C. Investigation of the Defect Density in Ultra-Thin Al2O3 Films Grown Using Atomic Layer Deposition. *Surf. Coat. Technol.* **2011**, *205*, 3334–3339, doi:10.1016/j.surfcoat.2010.12.001.
